# Study protocol: to investigate effects of highly specialized rehabilitation for patients with multiple sclerosis. A randomized controlled trial of a personalized, multidisciplinary intervention

**DOI:** 10.1186/1472-6963-12-306

**Published:** 2012-09-06

**Authors:** Jan Sørensen, Anne Lee, Brita Løvendahl, Michael Nørgaard, Jette Bay, Peter Vestergaard Rasmussen, Finn Boesen

**Affiliations:** 1 Centre for Applied Health Services Research and Technology Assessment, University of Southern Denmark, J. B. Winsløws Vej 9B, 1, 5000, Odense, Denmark; 2 Sclerosis Hospitals in Denmark, Haslev and Ry, Ringstedvej 106, 4690, Haslev, Denmark; 3 The Danish Multiple Sclerosis Society, Mosedalvej 15, 2500, Valby, Denmark; 4 Department of Clinical Medicine – The Department of Neurology, Aarhus University, Nørrebrogade 44, 8000, Aarhus, Denmark

## Abstract

**Background:**

Multiple sclerosis (MS) is a complex, chronic and progressive disease and rehabilitation services can provide important support to patients. Few MS rehabilitation programs have been shown to provide health improvements to patients in a cost-effective manner. The objective of this study is to assess the effects in terms of changes measured by a variety of standardized quality of life, mastery, coping, compliance and individual goal-related endpoints. This combination provides the basis for analyzing the complexity of MS and outcomes of a personalized rehabilitation.

**Methods/Design:**

Patients with MS referred to hospital rehabilitation services will be randomized to either early admission (within two months) or usual admission (after an average waiting time of eight months). They will complete a battery of standardized health outcome instruments prior to randomization, and again six and twelve months after randomization, and a battery of goal-related outcome measures at admission and discharge, and again one, six and twelve months after randomization.

**Discussion:**

The results of the study are expected to contribute to further development of MS rehabilitation services and to discussions about the design and content of such services. The results will also provide additional information to health authorities responsible for providing and financing rehabilitation services.

**Trial registration:**

Current Controlled Trials (ISRCTN05245917)

## Background

Multiple Sclerosis (MS) is a complex, chronic and progressive disease with currently more than 10.000 Danes diagnosed [1]. Patient rehabilitation is necessarily also complex, requiring a personalized, multidisciplinary and highly specialized effort [2,3].

Modern immune drug therapy has not convincingly delayed the progressive neurodegenerative process in MS [4] and it remains unclear to what extent medical treatment can prevent permanent loss of functional abilities. Rehabilitation services will thus continue to be as important as pharmacological interventions for patients with MS. As such, they should be designed according to guidelines based on high quality evidence and assessed using standard evaluative methods.

Personalized rehabilitation is based on the premise that every individual is unique in terms of biology, personality, psychology, physical and social ability in combination with a unique life history formed by social circumstances, daily activities and life style. The objective of personalized rehabilitation is to improve patients’ functional status and to improve extroversion, activity and participation by strengthening the individual’s motivation and ability to cope with the disease and related challenges for living an active life and to be active with their own rehabilitation. Personal rehabilitation takes place during meetings between active, well-prepared patients and rehabilitation teams.

MS rehabilitation is a process that from the beginning is matched to the patient's individual needs and desires and includes active involvement of both the patient and relatives. The patient has a personal coach and a trained specialist as sparring partners throughout the rehabilitation process and these provide guidance to the rehabilitation team composed to support the patients’ needs and goals.

Through coaching patient can be supported to set goals for the rehabilitation. Such goals can be two-dimensional: to achieve at positive changes in the person's life and to be realistic about achievable goals for the rehabilitation process. When the goals of the rehabilitation are determined, a unique personalized program may be developed so that the content and intensity match the patients’ needs, opportunities and constraints. The rehabilitation process can therefore be characterized as shared decision-making between the patient (and relatives) and the supervisor and the rehabilitation team with regular assessment of content in the rehabilitation program and progression in relation to results.

The Sclerosis Hospitals at Haslev and Ry were established by the Multiple Sclerosis Society in 1960 to offer specialized treatment to people with MS in Denmark. Since the early 1990s the hospitals have had annual operating agreements with the Danish health authorities and today are highly specialized hospitals with recent quality accreditation from the Danish Quality Model for Hospitals [5].

The Danish Multiple Sclerosis Hospitals provide personalized rehabilitation through a four-week inpatient program after referral by general practitioner or neurologist. A highly qualified and experienced multidisciplinary team collaborates with the patient in assessing and providing a personal program based on the individual patient’s needs. The program includes a range of training, treatment and education focusing both on the whole person and on specific conditions caused by MS. The goal of the rehabilitation program is to improve the patients' functional capacity in all relevant dimensions: participation and activity; physical, social and psychological functioning, including the patients’ ability to cope with the disease and its symptoms and to be active in their own rehabilitation. This personalized rehabilitation program can be seen as a paradigm shift in relation to an earlier and more uniform rehabilitation program.

### Evidence for MS rehabilitation

A recently updated version (April 2011) of a Cochrane review [6] identified ten studies of varying quality and concluded that although MS rehabilitation may have limited effect on body functions, short-term improvement in activity and participation could be observed. Another Cochrane review suggested that exercise therapy might be beneficial for muscle power function, exercise tolerance, mobility-related activities and mood in patients with MS, although no clear effects could be identified in relation to fatigue or perception of handicap [7].

The efficacy and cost-effectiveness of the Sclerosis Hospitals rehabilitation program for MS patients were investigated in a longitudinal study [8], in which participants completed a single outcome instrument (FAMS questionnaire) to measure quality of life at referral, admission, discharge and up to five months after discharge. The study showed an increase in the FAMS score from referral and admission to discharge and thereby identified a statistically significant improvement in quality of life. Although the FAMS scores had decreased five months after discharge, there was still a statistically significant improvement compared with the score at referral. The study had a number of design issues that complicated assessment of the rehabilitation effort. As the study had no control group, part of the observed effect may have been due to factors other than the rehabilitation program. The considerable variation in participants' personal and disease-related factors could also have influenced the observed effects.

### Study objectives

The aim of the planned study is to document the effects of a highly personalized rehabilitation program for MS patients as provided by the Sclerosis hospitals using a randomized controlled study design and standard generic and disease-specific outcome measures. The objective is to assess the program’s effect on patients in terms of changes in disease-specific quality of life and to answer the following questions:

To what extent are the observed effects maintained six and twelve months after discharge?

How do different dimensions of quality of life, functional ability, mastery, coping, compliance and specific measurements of sleep, daily activities, gait, balance and activity limitations correlate?

What individual goals are set before the start of the rehabilitation?

What are the central areas and priorities of the personalized rehabilitation program?

What specific and measurable actions are included in the personalized rehabilitation program?

What effect does the rehabilitation process have on individual patient’s goals and priorities at discharge?

To what extent do patients maintain coping, one month after discharge?

What resource use and costs occur for patients and their relatives, and for the rest of the health care system?

What is the relationship between the obtained effects and costs?

## Methods and design

The study is designed as a pragmatic clinical trial [9] of a complex intervention with personalized outcomes [10]. It attempts to maximize external validity by incorporating important features of the rehabilitation intervention in real-life settings and allowing individual participant-selected treatment goals and personalized outcomes.

**Figure 1:**
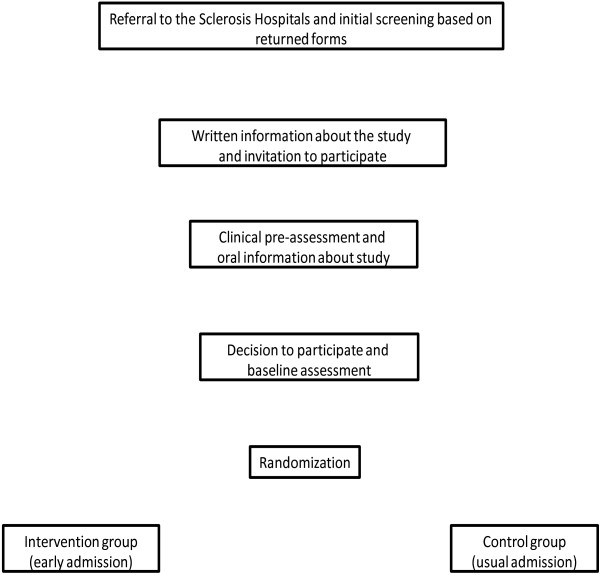
Patient selection for a randomized study of a MS rehabilitation program.

The study design (Figure 1) is a two-hospital controlled trial of the rehabilitation intervention with randomization to either early admission (‘intervention group’; admission within two months after referral and clinical prescreening) or usual admission (‘control group’; admission approximately eight months after referral to the hospital and clinical prescreening). Any differences between these two groups on observed measures between baseline and six-month assessments will be considered as effects of the rehabilitation program. As the control-group patients are admitted to the hospitals after approximately eight months from referral and prescreening, they also contribute with data to the effect of personalized rehabilitation. To observe long-term effects, data collection will be repeated after twelve months.

### Participants

Study participants will be recruited among all patients referred to the four-week individual rehabilitation program during March 2012 to June 2013. Patients referred for shorter rehabilitation programs or theme courses will not be eligible for the study.

Participants must meet the following inclusion criteria: aged between 18 and 65 years; diagnosis of MS (relapsing remitting, primary or secondary progressive MS); Expanded Disability Status Scale (EDSS) score ≤ 7.5 [11]; can use a personal computer or has a support person/relative who can; ability to read and understand sufficient Danish to understand instructions both orally and in writing; completion of written informed consent.

The following exclusion criteria will apply: less than six months between diagnosis of MS and referral to the program; disease relapse within three months of the neurological appraisal; recipient of sclerosis-specific hospital-based rehabilitation within the last six months; cognition score (KFS) > 2 or cognitive limitations which hinder completion of self-reported questionnaires and/or informed consent; severe depression; severe heart or lung disease; drug or alcohol abuse; any other illness that can impede participation in the study. If participants develop a disease that might impair their participation in the study they will be excluded from the study at that time.

The final decision on participant inclusion or exclusion will be based on the physician/neurologist assessment.

### The study intervention

The purpose of the rehabilitation program is to provide a personalized approach that allows the patient to develop knowledge and understanding of the disease and to maintain or develop the necessary resources and abilities to manage their life situation.

The stages of the study intervention are described below and include: initial screening; clinical examination prior to inclusion; randomization; a preparation day; four-week hospitalization; six and twelve months follow-up.

### Initial study screening

Patients referred to the hospitals are routinely sent a brief health screening questionnaire that upon return is assessed by the admission nurse to exclude patients who have a dependency level requiring intensive care and thus lack the rehabilitation potential defined by the inclusion criteria (equivalent to a EDSS score >7.5). The screening includes two basic models: factor evaluation and prototype evaluation, and includes assessment of physical and cognitive function/limitations (modified Barthel-100 index) [12-14]. The screening can be conducted as a home visit if necessary. Proposals for patient exclusion from the study will be discussed with a neurologist. The initial screening does not exclude wheelchair users or patients who need help from others to complete the questionnaire.

### Clinical examination prior to study inclusion

Patients who fulfill the initial screening criteria will receive by ordinary mail a study information pack including detailed information about the study and patients’ rights in a health science research project. Patients willing to participate in the study should respond within 10 days by phone or mail.

Potential participants will be invited to an ambulatory consultation with a neurologist. The consultation will include standard neurological examination, screening for severe depression [15] and a walking test. The neurologist completes a screening form covering information about the patient's previous and current medical treatment and information relevant to the in- and exclusion criteria. Patients will receive additional oral information about the study and a final decision about participation is taken. Patients who are not included in the study will continue in the usual rehabilitation program. Information on fulfillment of the in- and exclusion criteria, and stated reasons for non-participation will be recorded. Patients who need time to make a final decision whether to participate in the study or not, should return a completed consent form within two days of the clinical examination.

### Randomization

Randomization will be performed by staff at the admitting hospital using specialized computer software. To ensure balance between different patient groups, the randomization will be stratified according to gender, age (four groups), MS type (three groups), duration of diagnosis (five groups), EDSS score (three groups), and whether or not the patient is admitted to the hospital for the first time (two groups).

### Preparation day

The preparation day will take place seven days prior to the scheduled hospitalization. The overall purpose of the rehabilitation program will be explained and the individual areas for rehabilitation efforts will be defined in a collaboration between the patient and an appointed supervisor who serves as both the patient’s coach and as an 'orchestra conductor' for the rehabilitation team during the entire hospitalization. Prior to this preparation day, the patient will complete a FAMS form [16], a questionnaire on diet, smoking, alcohol and exercise, and indicate which of five focus areas is most important for him/her:

Energy level, including fatigue, scheduling, breaks and structure

Cognitive function, including memory, concentration and insight

Physical function, including walking, balance, endurance, strength and mobility

Psychological well-being, including strengthening coping mechanism, confidence, self-care, cognition and adjustment processes

Personal needs, including transferring, urination, toilet visits, bathing and medication.

The patient will also complete Form one in MYCaW [17,18]. The conversation follows clinical guidelines for target setting content and includes COPM [19-22] as identification and qualification of the decision basis for the objective. The conversation leads to identification of the primary goal within the five main areas (see above) and specific subsidiary goals and described cf. International Classification of Functioning, Disability and Health (ICF) classification of activity or participation level, supplemented by specific milestones [23].

### Four-week hospitalization

A hospitalization period of 20 days is organized as four weeks of continuous hospitalization. A team of relevant professionals is composed based on the assessment of the patient's needs and may include neuropsychologist, clinical psychologist, occupational therapist, physiotherapist, nutritional therapist or dietitian, nurse, healthcare assistant, nursing assistant, social worker. The neurologist will carry out the primary standard neurological examination and serve as consultant for the patient and supervisor for the team. Symptomatic drug therapy is organized or administered after the usual guidelines and the neurologist’s judgment.

The patient’s supervisor is responsible for the content of the patient's program and should oversee that it contributes to the work of the team and patient, works towards the agreed objectives and milestones and that the necessary adjustments are made during hospitalization. The supervisor and the patient continuously evaluate the program, for example by weekly conversations, and at discharge. According to patients’ wish, relatives and other team members may participate in one or more of the conversations.

During the rehabilitation period process data are collected on selected specific rehabilitation interventions, their nature, number, duration and medication.

### Outcome measures

Patient outcome will be measured by a variety of standardized quality of life (QoL), mastery and individual goal-related endpoints. A combination providing the basis for analyzing the complexity of MS and outcomes of a personalized rehabilitation.

### Standard endpoints and associated measurement instruments

Disease-specific quality of life: Multiple Sclerosis Impact Scale version 2 (MSIS-29) [24,25] and Functional Assessment of Multiple Sclerosis (FAMS) [16,26]

Mastery: Health Education Impact Questionnaire (heiQ) [27]

Generic Quality of Life: EQ-5D [28-30] and 15D [31]

Expanded Disability Status Scale score (EDSS) [11]

Fatigue: Modified Fatigue Impact Scale (MFIS) ) [32,33] (also used as individual endpoint)

Sleep: Epworth Sleepness Scale (ESS) [34], Pittsburghs Sleep Quality Index (PSQI) [35-38] (also used as individual endpoint)

Self-assessment of the severity of two main self-selected problems for which the patient seeks help and general feeling of well-being (MYCaW) [17,18]

Resource consumption and costs for patients and their relatives, and for the rest of health care service

### Individual goal-related endpoints and associated measurement instruments

After randomization, each participant will attend a preparation day, when individual goals and endpoints for the rehabilitation program will be determined according to the following areas:

Energy level: Modified Fatigue Impact Scale (MFIS) [32], Epworth Sleepness Scale (ESS) [34], Pittsburghs Sleep Quality Index (PSQI) [35-38]

Cognitive function: Modified Fatigue Impact Scale (MFIS) [32]

Physical function: Six-minute walk test (6MWT) [39-41], Multiple Sclerosis Walking Scale [42-45], Dynamic Gait Index (DGI) [46,47], Five Times Sit-To-Stand Test (5SST) [48,49], 9-Hole Peg Test (9-HPT) [50-52] and Six Spot Step Test (SSST) [53,54]

Psychological well-being and: Multiple Sclerosis Self-Efficacy Scale (MSSE) [55-57] and Coping Self-efficacy Scale (CSE) [58-60]

Personal needs: Barthel-100 index [12-14,61]

These questionnaires and physical tests are selected to capture changes in individual goals.

The Mastery (heiQ) and QoL (FAMS) are the only tests to be performed also one month after discharge in order to assess any changes in mastery and QoL shortly after returning home.

The individually chosen tests will be supplemented by Assessment of Motor and Process Skills (AMPS) [62,63] that include measurements of efficiency, safety, autonomy and effort in motor and process skills. This is relevant for measurements of changes in all five focus areas.

Physical tests will be performed at the clinical examination prior to inclusion, at the preparation day, at discharge and at 6 and 12 months follow-up. If the patient has received an aid during the rehabilitation program, the discharge tests will be performed both with and without aids. For practical reasons and to avoid patient burden, it is probably not possible to conduct all the physical tests for a participant during one morning session. Some tests may thus be delayed (after a rest or the following day). Such an approach is not expected to influence the validity of the test results [64]. However as patients are typically tested in the afternoon at inclusion, they will also be retested in the afternoon at discharge to avoid any effects of time differences.

### Data collection

The data collection is planned as outlined in Table 1. Measurement times for the early admission group include baseline (Time A1), preparation day (Time A2), day of discharge (Time A3), one-month follow-up after discharge (Time A4), six-month follow-up after baseline (Time A5) and twelve-month follow-up after baseline (Time A6).

**Table 1:** Time points for data collection during the study

	**Intervention (early admission)**	**Control (usual admission)**
Baseline	Time A1	Time B1
6 months after baseline		Time B2
Preparation day	Time A2	Time B3
Day of discharge	Time A3	Time B4
1 months after discharge	Time A4	Time B5
6 months after baseline	Time A5	
12 months after baseline	Time A6	Time B6

Similarly, the six measurement times for the usual admission group are baseline (Time B1), six-month follow-up after baseline (Time B2 - acts as the control measurement – no intervention), the preparation day (Time B3), day of discharge (Time B4), one-month follow-up after discharge (Time B5) and twelve-month follow-up after baseline (Time B6).

Collection of patient-reported outcomes will mainly be based on online versions of the questionnaires, but questionnaires administered at the preparation day and at discharge will be in paper versions (Table 2). Patients who have completed the consent form will be assigned a unique study identification number and a login code to the web-based data collection system SurveyXact (http://www.surveyXact.dk). This password provides access to the questionnaire to be answered in accordance with the measurement time.

**Table 2:** Questionnaires and assessments in the study

**Instruments**	**A1/B1**	**B2**	**A2/B3**	**A3/B4**	**A4/B5**	**A5**	**A6/B6**
	**(baseline)**	**(6 mths after baseline)**	**(preparation day)**	**(day of discharge)**	**(1 mths after discharge)**	**(6 mths after baseline)**	**(12 mths after baseline)**
Personal characteristics							
Initial assessments	x						
Subsequent assessments		x				x	x
Standard outcomes							
MSIS-29	x	x		x		x	x
FAMS	x	x		x	x	x	x
heiQ	x	x			x	x	x
EQ-VAS	x	x		x		x	x
EQ-5D/15D	x	x				x	x
EDSS	x	x				x	x
MFIS	x	x				x	x
ESS/PSQ	x	x				x	x
MYCaW			x	x		x	x
Walking distance test	x	x				x	x
Goal-related outcomes							
Energy-level			x	x		x	x
Cognitive function			x	x		x	x
Physical function			x	x		x	x
Psychological well-being			x	x		x	x
Personal needs			x	x		x	x
Motor and Process Skills			x	x		x	x
Services used during admission							
Use of different services				x			

### Pilot study for data collection

In November 2011 a pilot study was conducted to test and adjust the screening and randomization processes, and to test the online survey facility. Data were collected for 8 patients in accordance with the protocol for the two groups. This pilot study identified a number of practical challenges that were addressed in the final study protocol. The target group was expanded from only patients who were referred for the first time to also include patients who had previously been admitted to the hospitals. The inclusion criteria were tightened to require that participants should be able to respond to the computerized questionnaires. A maximum age of 65 years and an additional exclusion criterion regarding other illnesses that could impede participation in the study were introduced. In addition, the screening for depression was changed to be part of the clinical assessment rather than being based on self-assessment.

### Register and economic data

In addition to data from questionnaires, data from routine administrative health registries will be obtained from the Sclerosis Hospitals, the national patient registry and the primary care registry (through the National Board of Health’s research service) and the registry of primary care prescription medicine. Data available in these registries include records of actual resource consumption (number of hospitalizations, outpatient visits and consultations) and costs in terms of service fees, fees paid to health care providers and pharmacy retail prices (AUP). Data on vital status will be obtained from the national mortality registry. These data will be obtained for the years 2010–14, i.e. for the two years prior to baseline and up to the 12 months of follow-up.

### Data analysis

#### Sample size calculation

Determination of the required sample size is based on known data from the previous FAMS study [8,26]. The FAMS total score ranges from 0-170 and the average score at referral was 103 (SD 27). Assuming that a 5% score change indicates a clinically significant change, then the mean score should change to more than 108 i.e. that a clinically significant change requires a 5-point change.

To test a 5-point difference between two groups with 5% statistical significance (alpha) and 90% power (beta), the calculated required number of participants in each group is 613 people. With 80% power the required number of participants is reduced to 458.

If we want to test a 10-point difference, there needs to be 154 people (assuming 90% power) or 115 people (80% power).

The current study participants are expected to be a more homogeneous group than in the previous study, thus the variation in FAMS score is likely to be reduced. If the standard deviation is assumed to be 20 (instead of 27), then 252 patients will be needed in each group to identify a 5% difference with 80% power.

Taking into account correlation between repeated measurements for the same individual and assuming that the correlation between two subsequent measurements is 0.8, a 5-point difference could be identified with 160 participants (90% power) or 120 participants (80% power) in each group.

Based on these assessments a reasonable sample size is considered to be around 200 participants in each of the two groups. The Sclerosis Hospitals expects to admit 1,200 people with multiple sclerosis in the period March 2012 through June 2013. An unknown number will be either excluded from the study or will decline to participate in the study. If an insufficient number of patients has been recruited at the end of the planned inclusion period, a (short) extension of the inclusion period will be possible.

### Statistical analyses

Analysis will be both descriptive and comparative.

### Descriptive analysis

Differences in personal and disease characteristics will be compared between participants completing the study, those who fulfill the inclusion criteria but decline participation (primary drop-out), and those who participate in parts of the study but for various reasons end participation (secondary drop-out). We will investigate for specific characteristics that distinguish drop-out individuals from those who complete the study.

The included study population will be described and the intervention and control groups will be compared for eventual significant differences in personal and disease characteristics. 'Intention to treat' analysis will be used for comparing the efficacy of the two groups and longitudinal random effects regression models (panel analysis) will be applied.

### Comparative analysis

The following comparative analysis will be conducted:

Difference from Time A1 to Time A5 for intervention group compared with the difference from Time B1 to Time B2 for control group (i.e. difference in outcomes for rehabilitation vs. no rehabilitation at 6-month follow-up)

Difference between Time A1 and Time B1 (i.e. difference between intervention and control group at baseline)

Difference between Time A1 and Times A5 and A6 (i.e. baseline vs. 6- and 12-month follow-up for intervention group)

Difference between Time B3 and Time B6 for control group (i.e. admission vs. 12-month follow-up).

Difference between A2 and A3 for intervention group (pre- vs. post-hospital rehabilitation)

Difference between B3 and B4 for control group (pre- vs. post-hospital rehabilitation).

Difference between Time A1 and Time A4 for intervention group A (baseline vs. one month after hospital rehabilitation)

Difference between Time B3 and Time B5 for control group (pre- vs. one month after hospital rehabilitation)

Changes in Mastery (heiQ) over time: Times A1, A4, A5 and A6 for intervention group compared with Times B1, B2, B5 and B6 for control group

Subgroup analyses will be performed on the individual focus areas, based on data from participants who have completed the goal-related questionnaires.

### Time scale

Patient recruitment will be ongoing and is expected to be completed by mid-2013. Data collection is expected to last a further twelve months until mid-2014. This period will be followed by a one-year period of data analysis and manuscript preparation.

### Blinding

It is not possible to blind participants in relation to which group they are randomized to. Similarly, it is not possible to blind the staff at the two hospitals regarding participation in the study and randomization group. Study participants will be admitted to the rehabilitation program concurrently with non-participants. This means that the intervention group and control group will be treated alongside patients outside the study.

### Ethics and permissions

Participation in the study will have no influence on the offer of rehabilitation or its content. Patients who do not wish to participate in the study will be treated as usual. All study participants will regardless of the randomization be offered rehabilitation.

The study is organized to meet the standards of the Declaration of Helsinki and the study protocol has been approved by the Research Ethics Committee of the Zealand Region (ref.no. 1-01-83-0002-07). The Danish Data Authority has granted permission to collect and store the required project information (ref. no. 2011-41-6751). The study is registered in the clinical trials database: http://www.controlled-trials.com/ISRCTN05245917/.

The study has obtained permissions to use the standardized outcome instruments.

## Discussion

This study is designed to investigate the effects of an inpatient rehabilitation program offered to individuals with multiple sclerosis. The results will contribute to further development of MS rehabilitation services and to discussions about the design and content of such service. The results will also provide additional information to health authorities responsible for providing and financing rehabilitation services.

To our knowledge this study will be one of the first randomized clinical trials to examine patient outcomes from a personalized rehabilitation program for MS. A wide variety of outcomes will be assessed immediately after discharge as well as one, six and twelve months after completion of the rehabilitation the program completion. A combination providing the basis for analyzing the complexity of MS and outcomes of a personalized rehabilitation. Cost-effectiveness will be assessed at twelve months.

## Competing interests

JS and AL are full-time employees of the University of Southern Denmark. The Danish Multiple Sclerosis Society has provided an unrestricted grant to the University as financial support for their involvement in this study. BL, MN, PVR and FB are employees of the Danish Multiple Sclerosis Hospitals and have managerial and clinical responsibility for providing the personalized rehabilitation services. JB is a member of the Danish Multiple Sclerosis Society’s management board, Chairman of the Society’s Health committee and member of the Sclerosis Hospitals’ management board.

## Authors’ contributions

The authors have jointly developed the protocol during long-term research collaborations. All authors have contributed to all elements of the study design, drafting and commenting of the manuscript. All authors have approved the final manuscript. JS assumes the role as guarantor.

## Pre-publication history

The pre-publication history for this paper can be accessed here:

http://www.biomedcentral.com/1472-6963/12/306/prepub
